# Biomarkers of Community-Acquired Pneumonia: A Key to Disease Diagnosis and Management

**DOI:** 10.1155/2019/1701276

**Published:** 2019-04-30

**Authors:** Elena N. Savvateeva, Alla Yu. Rubina, Dmitry A. Gryadunov

**Affiliations:** Engelhardt Institute of Molecular Biology, Russian Academy of Sciences, Vavilova Str. 32, 119991 Moscow, Russia

## Abstract

Community-acquired pneumonia (CAP) is a dangerous disease caused by a spectrum of bacterial and viral pathogens. The choice of specific therapy and the need for hospitalization or transfer to the intensive care unit are determined by the causative agent and disease severity. The microbiological analysis of sputum largely depends on the quality of the material obtained. The prediction of severity and the duration of therapy are determined individually, and existing prognostic scales are used generally. This review examines the possibilities of using specific serological biomarkers to detect the bacterial or viral aetiology of CAP and to assess disease severity. Particular emphasis is placed on the use of biomarker signatures and the discovery of biomarker candidates for a single multiplex analysis.

## 1. Introduction

Community-acquired pneumonia (CAP) is one of the most common infectious diseases and an important cause of death in children under 5 years old in developing countries and in adults over 65 in developed countries. Bacterial and viral agents are the main causes of pneumonia [1]. Fungal and parasitic lung infections are less-common causes [2, 3].

Pneumonia can result from the effect of a respiratory virus on the lungs that leads to both primary viral pneumonia and pneumonia with a secondary bacterial aetiology, as well as to later bacterial complications of the respiratory tract viral illness. Some patients develop a mixed infection with a viral–bacterial aetiology. In addition, CAP can be caused by several pathogens simultaneously [4]. Patients with immunosuppression, those with concomitant chronic obstructive pulmonary disease or chronic asthma, and those with pulmonary tuberculosis should be distinguished as separate groups [5, 6].

The range of bacterial pathogens that cause inflammation in the lungs is quite extensive [1]. The largest group is represented by extracellular bacteria, such as* Streptococcus pneumoniae*,* Haemophilus influenzae*, and* Staphylococcus aureus*. Another group includes the “atypical” intracellular bacteria, such as* Mycoplasma pneumoniae*,* Chlamydophila pneumoniae*, and* Legionella pneumophila,* which are difficult to identify using traditional culture methods [7]. No clinical features exist that allow intracellular and extracellular pathogens in pneumonia to be discerned, although extrapulmonary manifestations are often associated with intracellular pathogens in CAP [8]. The proportion of severe pneumonia cases involving “atypical” bacteria is estimated to range from 1 to 7% [7]. Moreover, coinfection with other pathogens is frequent in severe CAP cases. A study by Cilloniz et al. [9], which included 362 adult patients with severe CAP, found that 10% of the cases with a defined microbial aetiology were caused by intracellular pathogens. Coinfection involving intracellular pathogens and other pathogens was observed in 30% of cases caused by intracellular pathogens.

Clearly, respiratory viruses can both cause pneumonia and predispose the patient to secondary infection with bacterial pathogens [10]. However, the interplay between the viruses, bacteria, and host during coinfection is incompletely studied [11]. The direct interaction of the viral protein with the bacterial agent appears to lead to increased bacterial virulence and poor clinical outcomes [12]. The viral agents most frequently identified in patients hospitalized with pneumonia are rhinovirus, influenza virus, respiratory syncytial virus (RSV), parainfluenza virus (PIV), and adenovirus [13]. However, it is important to carefully assess the contribution of various agents to the incidence of pneumonia, because the causative pathogen cannot be detected in more than half of patients hospitalized with pneumonia [14]. The discovery of new viruses associated with the development of pneumonia may clarify the aetiology of the disease [15].

Despite technological advances in molecular diagnostics, identifying the cause of pneumonia remains a challenge [16]. Recent studies have shown that the proportion of primary viral pneumonia among all cases of CAP is underestimated and is comparable to the proportion of bacterial pneumonia [17, 18]. However, there are no clinical guidelines for the differential diagnosis of primary viral and bacterial pneumonia and no consensus concerning the necessity of antimicrobial therapy for patients with obvious primary viral pneumonia.

Due to the wide range of possible aetiological agents and difficulties in obtaining representative samples, limitations in the detection of the specific pathogen responsible for CAP remain unresolved. Murdoch and coauthors [19] consider the limitations faced by researchers solving the problem of pneumonia aetiology. The first limitation is the quality of the clinical sample obtained from the patient. The detection of known pathogens in good-quality samples collected directly from the lower respiratory tract would provide evidence for the microbial aetiology of the pneumonia, especially that caused by microorganisms that usually do not colonize the upper respiratory tract. However, sample collection from the lower respiratory tract as an infection source can be difficult, creating a fundamental problem in establishing pneumonia aetiology. Although state-of-the-art diagnostic tests claim ultra-high sensitivity, they have limitations, because appropriate clinical specimens cannot always be obtained from a patient. In addition, a dilemma arises with pneumonia pathogens that can colonize the upper respiratory tract of healthy people (for example,* S. pneumoniae*) [20]. Does evidence of such pathogens in the sample indicate the presence of infection or contamination by a colonizing microorganism? If a pathogen such as rhinovirus that replicates in the upper respiratory tract and causes a spectrum of diseases is detected in a respiratory specimen, the question arises as to whether this pathogen is the cause of the pneumonia or an accidental finding from a recent upper respiratory tract infection. The difficulty in understanding the true cause of the pneumonia becomes apparent when more than one pathogen is found in a single sample. We thus need a more reliable and unambiguous test for the differential diagnosis of viral and bacterial pneumonia. In this regard, the study of the biomarkers that enter a patient's bloodstream during the onset and course of the disease seems to be a promising direction without the methodological limitations described above [21].

**Figure 1:**
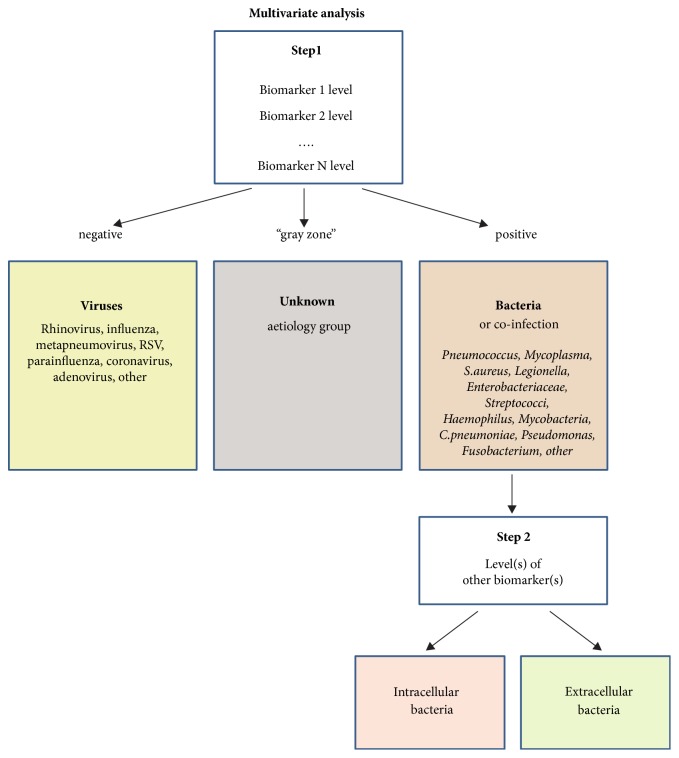
Simplified biomarker-based algorithm for establishing the aetiology of community-acquired pneumonia.

An ideal biomarker, biomarker combination or biomarker signature should not only exclude the bacterial aetiology of the disease but also be able to divide patients with bacterial pneumonia into subgroups that require different treatment strategies (Figure 1).

In this review, we consider the possibility of using biomarkers to identify the bacterial or viral aetiology of CAP and predict the severity of the disease. Both conventional inflammatory biomarkers and potential markers of the anti-inflammatory response to viral and bacterial invasion are discussed. Particular emphasis is placed on the use of biomarker signatures as well as on the search for candidate biomarkers for application in a single multiplex analysis.

## 2. Revealing the Aetiology of CAP

Studies of the diagnostic application of biomarkers in CAP have shown that proteins of the acute phase of inflammation and signaling molecules can be potential indicators of the onset and course of the disease [22, 23]. The first line of defence in the lungs is the airway epithelial cells, tissue-resident alveolar macrophages and monocytes circulating in the bloodstream. The ability of alveolar macrophages to produce cytokines is currently believed to be key for the initiation of immune responses in the lungs [24]. Determination of the cytokine regulatory network structure in various disease aetiologies may elucidate the systemic inflammatory response in pulmonary pathologies. Although not all candidate biomarkers have been fully investigated, procalcitonin (PCT) and C-reactive protein (CRP) are the most-studied biomarkers used in clinical practice for CAP management. The most widely studied area is measurement of the PCT and CRP levels in patients with pneumonia among those infected with the 2009 H1N1 influenza virus.

After the 2009 pandemic of the highly virulent influenza A H1N1 virus, a series of retrospective studies focusing on biomarkers as an additional criterion for discriminating between primary viral and bacterial pneumonia were published. Australian scientists studied a group of patients with CAP who arrived in the intensive care unit during the epidemic of influenza A/H1N1/09 [25]. The authors found that a bacterial infection alone or in combination with influenza virus infection is unlikely when the PCT value is low, especially in combination with a low CRP level. In combination with clinical symptoms, low levels of PCT and CRP potentially identified a group of patients for whom antibiotic therapy was not rational.

Wu et al. [26] performed a meta-analysis of six original articles, which included the results of a study of 518 patients infected with influenza A virus. The authors concluded that the PCT level for the differential diagnosis of mixed bacterial and influenza pneumonia had a high sensitivity (84%) but a low specificity (64%) for the detection of secondary bacterial infections among patients with influenza.

Pfister et al. [27] studied five original articles describing, in total, 161 patients admitted to the intensive care unit with suspected pneumonia associated with influenza A H1N1 virus infection. The PCT levels were significantly higher in patients with bacterial pneumonia than in patients with viral pneumonia. In this cohort, the PCT level was a sensitive marker (sensitivity 85.5%, negative predictive value (NPV) = 82.2%) and exceeded the diagnostic capabilities of CRP to identify bacterial pneumonia. The discriminatory power of CRP, as assessed by the area under the curve (AUC) (0.64), was lower than that of PCT (AUC = 0.76). However, the NPV of PCT was insufficient for its use as a stand-alone marker for withholding antibiotic therapy in such critically ill patients.

PCT and CRP have also been studied as biomarkers for distinguishing cases of pneumonia caused by intra- and extracellular bacteria. Kruger et al. [28] studied PCT in a population of 1337 inpatients and outpatients with CAP, including 472 patients with an identified pathogen. The authors demonstrated that the PCT, CRP, and white blood cell (WBC) values were significantly higher in CAP with a typical bacterial aetiology than in atypical bacterial or viral pneumonia. However, these inflammatory markers did not allow the prediction of an individual microbial aetiology of CAP.

In another work, Self et al. evaluated the association of the serum PCT concentration with the aetiology of pneumonia in a large cohort (n=1735) of hospitalized adults with CAP who underwent systematic testing for viruses and bacteria [29]. Despite thorough pathogen examinations, which included both traditional culture-based and serological tests, as well as PCR-based tests, the pathogens in 62% of the patients samples could not be identified. The authors found that no PCT threshold perfectly discriminated between bacteria and viruses. However, higher serum PCT concentrations showed a good correlation with an increased probability of a bacterial pathogen. Interestingly, the PCT values in cases of CAP with atypical bacteria were more similar to those of CAP with viruses than with typical bacteria. This was particularly true for* Mycoplasma* and* Chlamydophila*, but not* Legionella*. While these results suggest that PCT is a better marker for typical bacteria than for the combined group of typical and atypical bacteria, this conclusion should be confirmed by future studies.

Considering the significance of the possible fatal error of a false diagnosis for patients with bacterial pneumonia, the role of PCT and CRP as independent biomarkers to exclude bacterial infection of the lower respiratory tract is limited. This finding has been confirmed by a recent study, in which the use of a PCT-guided antibiotic prescription did not result in less exposure to antibiotics than did usual care among patients presenting to the emergency department with a suspected lower respiratory tract infection [30].

Along with PCT and CRP, a number of studies have considered the possibility of using other proteins involved in the acute phase of inflammation, e.g., lipopolysaccharide-binding protein (LBP), as well as cytokines to establish the aetiology of CAP. However, the data obtained in recent works are contradictory. An important limitation for researchers is the short period of cytokine life in the blood. In addition, the time elapsed from the onset of disease to the moment of blood sample collection contributes to the results of the analysis.

Hence, Kim et al. [31] studied hospitalized paediatric patients with influenza virus infection and pneumonia (N = 57) and found that the concentrations of interferon alpha (IFN-*α*), IL-6, and interferon *γ*-induced protein 10 (IP-10) were higher in children with both influenza A/H1N1 and pneumonia than in patients with pneumonia without H1N1 infection. In total, 10 cytokines (IFN-*α*, IFN-*γ*, IL-1*β*, IL-4, IL-6, IL-10, IL-17, IP-10, macrophage inflammatory protein (MIP)-1*α*, and tumour necrosis factor alpha (TNF-*α*)) were analysed.

Zobel et al. [32], in their study of 1000 adult patients, showed that the levels of IL-6, IL-10, and LBP were increased significantly in pneumonia. Higher concentrations of cytokines were detected in patients with typical bacterial infections caused by* S. pneumoniae*,* H. influenzae*, etc. Moreover, the LBP level was the best biomarker for differentiating patients with typical bacterial infections from those with atypical bacterial infections with* M. pneumoniae* and* Legionella spp*.

Menendez et al. [33] reported a cytokine response study in CAP. In addition to the PCT and CRP concentrations, the levels of TNF-*α*, IL-1*β*, IL-6, IL-8, and IL-10 were determined in 658 hospitalized patients. The data indicated significant differences in the cytokine levels depending on the aetiology of pneumonia: decreased IL-6 for atypical bacteria, increased IL-10 for viral, increased IL-8 for* Enterobacter *spp., and increased TNF-*α* for* L. pneumophila*. Notably, 57% of the patients in this study had pneumonia of an unknown aetiology.

However, a recent study published by Siljan et al. [34] of 247 patients hospitalized with CAP did not confirm the possibility of using cytokine levels to identify the aetiology of pneumonia. The concentrations of the terminal complement complex (TCC) and plasma cytokines were measured within 48 hours after hospitalization, at clinical stabilization and after six weeks of observation of the patient. The cytokine panel included 27 interleukins, chemokines, and growth factors. The level of most cytokines was higher during hospitalization than either at clinical stabilization or after six weeks. However, the cytokine response in the groups of patients with different aetiologies of pneumonia (bacterial, viral, viral–bacterial, or unidentified) was similar.

A promising research direction is the discovery of signatures for the rapid identification of the specific causative pathogen of pneumonia; the work by Strehlitz published in 2018 is a striking example [35]. The strategy for the empirical use of antimicrobials is inappropriate for CAP caused by methicillin-resistant* Staphylococcus aureus*. The authors performed a transcriptional analysis of lung tissue and blood samples from mice infected with* S. pneumoniae* and* S. aureus* and found a reliable signature associated with pneumococcal pneumonia. However, this signature was missed in mice infected with* S. aureus*.* S. pneumoniae* was demonstrated to induce a significantly stronger interferon response (IFN-*β*, IFN-*γ*, CXCL9, and CXCL10) than* S. aureus* in a mouse model. Moreover, a predictive model based on a combination of the CXCL9 (MIG) and CXCL10 (IP-10) levels in serum was validated in an independent cohort of mice and selected as the best model; this model included a minimal set of biomarkers and enabled the identification of infections caused by* S. pneumoniae* or* S. aureus*. It should be noted that although mouse models are a convenient tool in immunological studies, there could be significant differences between the responses of humans and mice to pathogens, and the results obtained using animal models cannot be translated directly to humans.

A brief overview of these studies is summarized in Table 1.

## 3. Biomarker Combinations and the Design of Signatures for CAP Aetiologies

None of the studies discussed above presented a biomarker-based test capable of distinguishing between bacterial and viral aetiologies of pneumonia with sufficient accuracy and specificity for clinical use. This section considers the possibilities of the combined use of biomarkers, or “signatures”, in revealing the CAP aetiology.

The use of a signature implies not only the simultaneous measurement of the levels of several markers but also the creation of a mathematical model based on the measured levels of the biomarkers for making a diagnosis.

The use of bioinformatics approaches allows the development of such a model considering the data obtained from different cohorts. Testing a mathematical model using a validation cohort enables a signature as an actual function of a model to be obtained and applied for the analysis of each sample. In the following text, the biomarker signatures will be designated with a “+” symbol. Signatures have been applied in a number of studies on the aetiology of CAP (Table 2).

Hence, Engelmann and colleagues [36] found that Myxovirus resistance protein 1 (MxA), which inhibits the early phases of viral replication by binding to viral ribonucleoproteins, is a specific marker of viral infection and can discriminate bacterial from viral infection with 96% sensitivity and 67% specificity (AUC = 0.89). However, the combined determination of the MxA + CRP levels allows identification of the CAP aetiology with improved diagnostic performance (AUC = 0.94).

Similar studies were conducted by Sambursky et al. [37]. These researchers proposed a combined test for the determination of both the MxA and CRP levels. Alone, neither MxA nor CRP was sensitive and specific enough to identify both viral and bacterial infections. Low cutoff values for CRP provided high sensitivity but low specificity, while high CRP cutoff level showed low sensitivity and high specificity for the detection of bacterial infection. The MxA level specifically identified viral infection but was not sensitive for the detection of bacterial infection. A test based on the simultaneous testing of two levels of CRP (20 mg/L and 65 mg/L), each evaluated in combination with the presence or absence of an increased level of MxA (> 40 ng/ml), was proposed. Among 54 patients, the MxA + CRP test correctly identified 92% of patients without infections, 80% of patients with confirmed bacterial infection, and 70% of patients with established viral infection.

Zhu et al. [38] proposed five biomarkers for distinguishing between bacterial and viral infections in the lower respiratory tract of children. In patients with bacterial infections, the levels of CRP, PCT, and interleukin 6 (IL-6), as well as the mean fluorescence intensity of CD35- and CD64-positive neutrophils, were significantly higher than the levels of these markers in patients with viral infections. The authors demonstrated that the discriminatory ability of the CRP + CD35 + CD64 (AUC = 0.973), CRP + CD35 (AUC = 0.963), and CRP + CD64 (AUC = 0.952) biomarker combinations to exclude bacterial infection was higher than that of any single biomarker (AUC = 0.804-0.904) or the CRP + PCT (AUC = 0.853) and CRP + IL-6 (AUC = 0.876) combinations.

One of the key works on the combined use of biomarkers to establish the aetiology of CAP in children is a study published in 2016 by Valim and coauthors [39]. In areas endemic for malaria, establishing the aetiology of pneumonia is difficult since malaria can also cause respiratory distress syndrome, which is clinically indistinguishable from bacterial pneumonia. The authors investigated candidate proteins in a multiplex immunoassay to determine biomarkers or biomarker combinations. The study included children hospitalized with suspected pneumonia (80 patients) and a control group of healthy children (10 patients). In accordance with a clinically confirmed diagnosis, the patients were divided into three aetiological groups: viral (30 patients), bacterial (23) and malarial (27). The levels of 99 cytokines, chemokines, other proteins, and metabolites were measured in plasma by HumanMAP multiplex immunoassays v. 1.6, v. 2.0, and v. 3.0 (Myriad RBM, Inc., USA). The patients were divided into two groups for screening potential biomarkers and for validation. Overall, the best individual biomarker for determining the aetiology of pneumonia was haptoglobin, with a sensitivity of 96% and a limited specificity of 68%. The best biomarker signature included haptoglobin in combination with the tumour necrosis factor receptor 2 (TNFR-2) (or the equivalent marker, IL-10) and tissue inhibitor metalloproteinase 1 (TIMP-1). The patients divided into bacterial and nonbacterial infection groups by haptoglobin level were further subdivided into subsets with viral and malarial disease aetiology. This signature allowed the correct classification of all 15 of the 15 patients with bacterial aetiology in the initial sample and 7 of the 8 patients in the validation sample. An alternate combination of markers with similar diagnostic accuracy was selected using regression models and included haptoglobin, IL-10, and creatine kinase MB isoenzyme (CK-MB).

One assay that integrates measurements of blood-borne host-proteins (tumour necrosis factor-related apoptosis-inducing ligand (TRAIL), which plays an important role in the regulation of both congenital and adaptive immune responses [40], IP-10, and CRP) was developed to differentiate bacterial from nonbacterial infections. After the screening of 600 potential biomarkers, the selection of biomarker signatures, and initial testing [41] and validation [42, 43], MeMed BV™ became the first assay to ensure the accurate differentiation of bacterial and viral infections in the blood. In 2017, van Houten et al. [44] performed a double-blind prospective study in a heterogeneous population and showed that compared with CRP and PCT, the combined CRP + TRAIL + IP-10 signature exhibited significantly improved diagnostic accuracy for lower respiratory tract bacterial infections in children. In terms of diagnostic performance, the triple-marker signature had a sensitivity of 87.8%, specificity of 93.0%, and positive predictive value (PPV) of 62.1% and NPV of 98.3%.

Recently, Ashkenazi-Hoffnung et al. [45] conducted a direct comparative study of the diagnostic efficacy of the combined CRP + TRAIL + IP-10 signature versus that of other candidate biomarkers. The study included 111 adults and 203 children with symptoms of a respiratory infection. The CRP + TRAIL + IP-10 signature was shown to have a significantly greater efficacy in the differential diagnosis of bacterial and viral infections than not only individual biomarkers, such as CRP, PCT, WBC, absolute neutrophil count (ANC), IL-6, and lipocalin-2 (NGAL/Lpc-2), but also currently used combinations of these biomarkers.

An essential obstacle of all studies evaluating the use of biomarkers to distinguish between bacterial and viral infections is the lack of objective clinical criteria that can be used to reliably discern between these aetiologies, and this factor limits the ability for positive and negative results of the analysis to be established. Microbiological approaches are used frequently as a reference. The disadvantages of these methods are described above; moreover, the microbiological confirmation of an aetiology can be obtained only in a limited number of cases.

Thus, cohort enrichment with easily diagnosable cases can occur, which can lead to overly optimistic diagnostic results. The analysis of nasopharyngeal smears by PCR to identify respiratory viral and bacterial pathogens is often available for only a part of the whole cohort, which can also lead to potential bias in the sampling of patients included in the study.

Typical problems in different work include the use of insufficiently large control samples and the collection of blood samples at different times from the onset of the disease. Despite some success, large-scale validation studies of detected biomarker signatures are necessary.

## 4. Severity Assessment in CAP

Currently, the mechanism of severe pneumonia is unclear and incompletely understood. The efforts of researchers are aimed at addressing gaps in identifying the causes of CAP severity in patients with similar histories. For example, mixed viral–bacterial infections may be associated with an increased risk of mortality [46]. However, as shown above, the exact establishment of the CAP aetiology is complicated.

An assessment of severity is carried out to decide whether a patient should be referred to a hospital or an intensive care unit or should undergo treatment outside the hospital. Current systems for assessing CAP severity include the CURB-65/CRB-65 score, Pneumonia Severity Index (PSI), and Severe Community-Acquired Pneumonia (SCAP) score, among other scores [47]. The most commonly used scores are the PSI, consisting of 20 clinical, laboratory, and radiological indicators; CRB-65 (impaired consciousness, a respiratory rate of ≥ 30 breaths/min, a blood pressure of ≤ 90/60 mm Hg, and an age of ≥ 65 years); and CURB (an increase in the urea nitrogen level of > 7 mmol/L replaces the age parameter) scores. The CRB-65 score is widely used; it is the easiest to calculate. However, these scales do not fully account for the functional status of the patient and the effect of associated diseases. For example, neither the CRB-65/CURB (a sensitivity of only 49%) nor PSI (a specificity of only 48%) score has sufficient accuracy to assess the necessity for transferring a patient to the intensive care unit [48]. The question remains as to whether biomarkers and biomarker combinations can help assess disease severity as well as predict the short-term and long-term survival of patients.

Numerous research groups have shown that the PCT level alone and the combined levels of PCT and CRP have prognostic value for estimating the risk of adult mortality from CAP (Table 3).

A significant increase in the average levels of PCT and CRP among nonsurviving patients at 28 days compared with those in survivors was demonstrated by Park et al. [49] and Kim et al. [50]. Moreover, the PCT level upon admission to hospitalization has been shown to predict the severity and outcome of CAP with high prognostic accuracy [51]. However, several contradictory results have been described for PCR and CRP as markers of CAP severity. Que et al. [52] studied patients admitted to the intensive care unit with severe CAP caused by* S. pneumoniae* and found no difference in the PCT level between patients with fatal outcomes and survivors. Moreover, a low initial CRP level in patients' serum was correlated with a fatal outcome. However, most clinicians characterize low CRP values as a less-pronounced response to systemic inflammation, which should reflect a favourable prognosis. Such discrepant results can be explained by the disparate populations included in the studies.

Several studies also investigated cytokine levels to predict the course of the disease. A study conducted by Haugen et al. [53] included 430 children aged 2 to 35 months with severe (N = 43) and mild (N = 387) CAP. Multiplex microbead-based immunoassays were used to determine the levels of 27 cytokines in plasma. The plasma concentrations of 11 inflammatory mediators with pro- and anti-inflammatory activity (IL-1, IL-4, IL-6, IL-8, IL-9, IL-15, eotaxin, bFGF, G-CSF, GM-CSF, and TNF-*α*) were higher in children with severe CAP than in children with mild pneumonia. The best correlation with disease severity was obtained for the combination of G-CSF and IL-6.

Wang et al. [54] examined the glycoprotein chitinase 3-like protein 1 (YKL-40, or CHI3L1) plasma level in adult patients with CAP (61 patients) and in a group of healthy donors (60 patients). YKL-40, or CHI3L1, is a proinflammatory cytokine belonging to the chitinase 18 family. Higher plasma levels of YKL-40 were detected in pneumonia patients than in the controls. Furthermore, the level of YKL-40 in plasma decreased significantly after treatment. The level of YKL-40 decreased significantly after treatment. Therefore, the plasma YKL-40 level can be used as prognostic serological marker of CAP. Later, another group of scientists included YKL-40 in a biomarker signature to predict the development of CAP [55]. In addition to the serum YKL-40 level, the levels of three potential biomarkers previously widely studied in chronic lung diseases, including surfactant protein D (SP-D), chemokine (C-C motif) ligand 18 (CCL18), and cancer antigen 15-3 (CA15-3), were measured. These four markers were also compared with the levels of the known inflammatory markers IL-6 and CRP. The study included 291 adult patients hospitalized with CAP, with 20 healthy non-smoking volunteers as the control group. Serum measurements were performed on the day of admission, on the second and fourth day of stay, and a minimum of 30 days later. The initial levels of YKL-40 and CCL18 were significantly higher in the patients than in the controls, but both markers were still elevated on the 30th day. This finding indicates continued cellular activity 30 days after the onset of pulmonary infection at a time when most patients were assumed to be in complete clinical remission. The SP-D, YKL-40, and CCL18 levels were higher in patients with severe pneumonia than in patients with nonsevere pneumonia, and the levels of YKL-40 and CCL18 were lower in patients with CAP caused by atypical bacteria than in patients with CAP caused by extracellular bacteria, for example,* S. pneumoniae*.

Interferon regulatory factor 5 (IRF5) plays an important role in regulating the induction of interferon genes by directly stimulating the expression of proinflammatory cytokines such as TNF-*α*, IL-6, IL-12, and IL-23. In addition, IRF5 suppresses the transcription of anti-inflammatory cytokines, for example, IL-10 [56]. Wang and coauthors [57] investigated the role of IRF5 in the regulation of immune responses in CAP patients and healthy donors. The expression of IRF5 mRNA and the levels of IL-6, IL-10, and IP-10/CXCL10 in the blood were shown to be correlated with the severity of inflammation in CAP patients; these levels can be prognostic biomarkers.

It should be noted that the use of interleukins as markers of diseases also imposes serious limitations on the efficacy and reproducibility of the analysis. The content of these cytokines in the circulating blood depends on many factors, and their levels are not stable either during the analysis or during storage. Moreover, a recent published study showed that the inflammatory response at the time of CAP diagnosis was influenced by the time since symptom onset. Méndez et al. [58] demonstrated that the time since the onset of symptoms to the diagnosis of CAP has a different effect on the systemic inflammatory profile of each biomarker. It was shown that the CRP level was significantly lower in patients presenting < 3 days since the onset of symptoms, while the PCT, IL-6, and IL-8 levels were already elevated. Another observation was that the PCT, IL-6, and IL-8 levels were significantly reduced after 3 days of symptoms.

## 5. Management of CAP Patients: Candidate Biomarkers for a Multiplex Assay

Changes in the levels of biomarkers specific to various organs may indicate organ dysfunction, the decompensation of associated diseases, and the development of complications. The coidentification of such biomarkers can be an auxiliary diagnostic tool for patient management.

Proadrenomedullin (MR-proADM), a more stable mid-regional fragment of the rapidly degrading active adrenomedullin (ADM) peptide, is a promising biomarker for inclusion in a signature predicting the severity and long-term adverse outcomes of CAP. In 2006, Christ-Crain et al. [59] showed that the level of MR-proADM, in contrast to the levels of CRP and leukocytes, increased with CAP severity. The initial MR-proADM levels were significantly higher in patients who died during treatment than in survivors. Later, Kruger et al. [60] included MR-proADM in a group of cardiovascular biomarker candidates for predicting short-term and long-term survival in CAP. The authors observed the management of 728 patients with CAP lasting over 180 days. The MR-proADM, atrial natriuretic peptide prohormone (MR-proANP), proarginine vasopressin (copeptin), proendothelin-1 (CT-proET-1), PCT, and CRP levels and the leukocyte number were measured, whereas disease severity was assessed using the CRB-65 scale, upon hospitalization. The level of MR-proADM, along with the combination of MR-proADM and CRB-65, showed the best diagnostic value in predicting short-term and long-term survival. The superiority of MR-proADM to other cardiovascular markers can be explained by the multifactorial function of ADM: unlike MR-proANP, copeptin, and proET-1, ADM has not only cardiovascular but also anti-inflammatory and antibacterial activity.

Later, Espana et al. [61] evaluated the possibility of using the biomarkers PCT, CRP and proADM in combination with the PSI, CURB-65, and SCAP scores for predicting adverse outcomes of patients with pneumonia. A prospective cohort study included 491 patients with CAP. The proADM level measured during hospitalization had a reliable correlation with the scores on the prognostic scales and improved the test accuracy. The proADM level in combination with the SCAP score showed the best diagnostic performance for predicting complications associated with pneumonia. Patients with any SCAP score (0, 1, or > 1) and a proADM level of <0.5 nmol/L did not require mechanical ventilation and transfer to the intensive care unit, nor did these patients develop complications or die. The combination of the proADM level and the SCAP score allowed the identification of a group potentially suitable for outpatient treatment.

Pneumonia can disrupt the integrity of the pulmonary endothelial barrier, which leads to acute respiratory system damage despite ongoing antimicrobial therapy. The Ang-1 and Ang-2 angiopoietins and their associated Tie2 receptors are involved in the regulation of vascular permeability and inflammation, but their role in pneumonia is unknown. Gutbier et al. [62] showed that Ang-1 and Ang-2 are useful biomarkers for predicting the severity of pneumonia and are potential therapeutic targets for the prevention of acute respiratory failure. A decrease in the Ang-1 level and an increase in the Ang-2 level in the serum of CAP patients compared with the Ang-1 and Ang-2 levels in the healthy control group were found. In addition, the Ang-2 levels differed significantly between surviving patients and those who died as a result of the disease. However, the Ang-2 levels were negatively correlated with the oxygenation index and positively correlated with various laboratory parameters, such as PCT, bilirubin, creatinine, and CRP.

A potential biomarker for inclusion in the diagnostic signature to assess the severity of pneumonia is the lipopolysaccharide (LPS) endotoxin, a major component of the cell wall of gram-negative bacteria. A significant increase in gram-negative bacterial infection among children with pneumonia forced scientists to search for new biomarkers for predicting the development of endotoxaemia, a severe destructive complication of CAP that often arises from infection of the lungs with gram-negative bacteria. Serum levels of LPS in children with pneumonia were shown to be significantly higher in patients with pneumonia caused by gram-negative bacteria than in controls [63].

The protective role of lipocalin-2 (NGAL/Lpc-2), produced by innate immune cells at an early stage of infection, is well known. The NGAL protein inhibits the growth of bacteria by binding iron [64]. In a paper by Huang [65], potential biomarkers of severe pneumonia in children were identified using proteomic analysis via mass spectrometry. NGAL/Lpc-2, CRP, and von Willebrand factor (vWF) levels were shown to be significantly increased in children with severe pneumonia. Moreover, NGAL is best biomarker of disease severity (sensitivity 72.3%, specificity 70.1%, AUC = 0.71). However, the method used to identify potential biomarkers did not allow the detection of proteins at concentrations below the nanogram per millilitre range, which excludes TNF-*α*, IL-6, and PCT, for example, although Bacci et al. [66] confirmed that the levels of inflammatory cytokines such as TNF-*α* and IL-6 were related to the prognosis of the disease. Later, the NGAL/Lpc-2 levels were studied by Kim and colleagues for the prediction of CAP severity and patient survival [67]; this study showed that the NGAL concentration was correlated with disease severity and that this marker could predict 30-day survival (AUC = 0.87).

Syndecan-4 (SYN4) is expressed in various cells, including alveolar macrophages and epithelial cells, and plays an important role in the inflammatory response in the lungs [68]. Nikaido et al. investigated the level of SYN4 in CAP patients [69], evaluating changes in the levels of the soluble form of SYN4 in serum during the course of therapy. Patients with a mild form of pneumonia had significantly higher levels of SYN4 than healthy volunteers. However, the level of SYN4 did not differ between patients with severe pneumonia and healthy volunteers. Despite the encouraging results, the use of NGAL/Lpc-2 and SYN4 as biomarkers during the course of pneumonia requires verification, since the results were contradictory when the levels of NGAL/Lpc-2 in combination with those of SYN4 were measured in children hospitalized with pneumonia [70].

The main limitation in the search and validation of new biomarkers is the cohort study designs used in various investigations. Biomarkers found in a uniform cohort are likely to perform well in cohorts similar to the original sample but less likely to perform well in other groups of patients. However, biomarkers detected in heterogeneous cohorts are likely to be successfully applied in a wider range of patients [71]. Combining biomarkers in a single multiplex analysis, namely, PCT, CRP, proADM, Ang-1, Ang-2, LPS, NGAL/Lpc-2, and SYN4 (Table 4), will allow validation studies with large cohorts to be carried out and clinically significant concentrations to be identified for use in clinical practice to assess the severity of CAP.

## 6. Conclusions

The incidence of CAP will undoubtedly increase in the next decade due to the ageing population and the subsequent increase in comorbidities. The microbiome of the upper respiratory tract is a factor ensuring lung health. Microbiome dysregulation is responsible for the growth and spread of potential pathogens, such as* S. pneumoniae*, which leads to acute respiratory infections, including pneumonia [72]. Why some people can maintain pulmonary equilibrium stay healthy, while others develop inflammation, is incompletely understood. Pneumonia has been suggested to be considered a chronic susceptibility to pathogens and not just an acute infection. Finding methods to measure and intervene in the chronic processes underlying pneumonia susceptibility could be the main goal in the near future [24]. The study of potential biomarkers for pneumonia susceptibility will define a new prevention strategy and will facilitate the timely initiation of therapy in vulnerable populations [73]. Thereby the analysis of biomarker levels in local inflammatory reactions in the lungs, especially in patients with mild disease, and the simultaneous characterization of the local and systemic inflammatory response are priority research areas [74, 75]. Such studies require accurate and reliable methods of multiplex analysis. Further evolution of proteomic technologies, mass spectrometry, multiplex assays based on microarrays [76], and micro- and nanoparticles will allow these tasks to be carried out [77].

Currently, there are no biomarker-based algorithms for establishing the aetiology of CAP. Although the use of PCT and CRP as biomarkers for discriminating bacterial infection has been discussed in various studies, these biomarkers cannot be used for true diagnosis of pneumonia. Furthermore, the data on the establishment of the CAP aetiology using the levels of signaling molecules are contradictory. Personalized patient treatment, including the search for biomarkers as disease precursors, is impossible without the use of biomarker combinations or signatures due to the low prognostic ability and high interindividual variation in single biomarkers. The immediate task is to conduct large-scale studies to validate biomarker signatures, allowing the establishment of the aetiology and the prediction of the course of pneumonia.

In the assessment of CAP severity, acute-phase proteins and signaling molecules in combination with CURB-65/CRB-65, PSI, and SCAP scores can effectively predict the development of pneumonia. The risk assessment of CAP complications can be improved by using biomarkers specific to organs affected by the disease. A signature including the PCT, CRP, proADM, Ang-1, Ang-2, LPS, NGAL/Lpc-2, and SYN4 levels would be useful for such an assessment. Because CAP is a rapidly developing disease, dynamic observation of the changes in the biomarker levels is of particular interest. The biomarker signature is only a fixed representation, a photograph, of the captured state; the results of subsequent analyses, even throughout a single day, can vary substantially. Clearly, the dynamic monitoring of changes in biomarker levels can be a useful auxiliary tool for the prompt selection of individual therapies for CAP.

## Figures and Tables

**Table 1:** Acute-phase proteins and signaling molecules in the estimation of CAP aetiologies.

Study [Ref.]	Study design	Patients	CAP aetiologies	Target biomarkers
Ingram et al., 2009 [25]	retrospective observational study in small cohort	25 adults	viral infection: 2009 H1N1 influenza (n = 16) bacterial/mixed infection (n = 9)	PCT, CRP

Wu et al., 2013 [26]	systematic reviews and meta-analyses	518 adults	viral infection: 2009 H1N1 influenza (n = 381) bacterial/mixed infection (n = 137)	PCT

Pfister et al., 2014 [27]	prospective cohort study systematic review and individual patient data meta-analysis	161 adults	viral infection: 2009 H1N1 influenza (n = 84) bacterial/mixed infection (n = 77)	PCT, CRP

Kruger et al., 2009 [28]	multicenter prospective cohort study CAPNETZ	1337 adults	unknown (n = 865) typical bacterial infection: (n = 185) atypical bacterial infection (n = 190) viral infection: Adenovirus, Influenza A, RS Virus, and Enterovirus (n = 39) mixed infections with two or more pathogens (n = 58)	PCT, CRP, WBC

Self et al., 2017 [29]	multicenter prospective active surveillance study	1735 adults	unknown (n = 1075) typical bacterial infection: (n = 169) atypical bacterial infection (n = 67) Mycobacteria/ Fungus (n = 15) viral infection: (n = 409), the most common pathogen were rhinovirus (n = 114)	PCT

Kim et al., 2011 [31]	prospective cohort study in small cohort	75 children	influenza H1N1 2009 virus infection -pneumonia (n = 57) pneumonia but without H1N1 infection (n = 18)	IFN-*α*, IFN-*γ*, IL-1*β*, IL-4, IL-6, IL-10, IL-17, IP-10, MIP-1*α*, TNF-*α*

Zobel et al., 2012 [32]	multicenter prospective cohort study CAPNETZ	1000 adults	unknown (n = 679) typical bacterial infections (n = 98) atypical bacterial infections (n = 155) viral infections: Adenovirus, Influenza A, Influenza B, RSV, and Enterovirus (n = 23) mixed infections (n = 45)	IL-6, IL-10, LBP

Menendez et al., 2012 [33]	prospective cohort study	685 adults	unknown (n = 390) causal diagnosis (n = 295): gram-positive cocci (n = 134), gram-negative bacilli (n = 69), atypical pathogens (n = 24), viral infections (n = 12)	PCT, CRP, IL-1 *β*, IL-6, IL-8, IL-10, TNF- *α*

Siljan et al., 2018 [34]	prospective cohort study	267 adults	unknown (n = 90) bacterial infection (n = 70) viral infection (n = 39) viral-bacterial infection (n = 48)	27 cytokines: IL-1*β*, IL-1ra, IL-2, IL-4, IL-5, IL-6, IL-7, IL-8/CXCL8, IL-9, IL-10, IL-12 (p70), IL-13, IL-15, IL-17A, bFGF, eotaxin/CCL11, G-CSF, GM-CSF, IFN-*γ*, IP-10/ CXCL10, MCP-1/CCL2, MIP-1*α*/CCL3, MIP-1*β*/CCL4, RANTES/CCL5, TNF-*α*, PDGF-BB and VEGF

Strehlitz et al, 2018 [35]	animal study	mice with *S. aureus* or *S. pneumoniae* respiratory infection	experimental murine models of bacterial pneumonia	mRNA levels of IFN-*β*, IFN-*γ*, CXCL9, CXCL10 IFN-*β*, IFN-*γ*, CXCL9/MIG, CXCL10/IP-10 Signature CXCL9+CXCL10 serum concentrations

**Table 2:** Biomarker combinations and signatures for CAP aetiologies.

Study [Ref.]	Study design	Patients	CAP aetiologies	Protein combination and signature
Engelmann et al., 2015 [36]	prospective multicenter cohort study	553 children	viral infections (n = 133) bacterial infections (n = 48) uninfected controls (n = 133)	MxA + CRP

Sambursky et al., 2015 [37]	prospective, single center, blinded, observational clinical trial	54 adults	viral infections: influenza A, influenza B, parainfluenza 2, parainfluenza 3 (n = 10) bacterial infections (n = 20) uninfected controls (n = 24)	MxA + CRP

Zhu et al., 2015 [38]	prospective cohort study	96 children	viral infections: Influenza virus, RSV, Human bocavirus, Parainfluenza virus, Adenovirus, Coronavirus, Influenza virus + RSV (n = 51) bacterial infections (n = 45)	CRP + CD35 + CD64, CRP + CD35, CRP + CD64

Valim et al., 2016 [39]	prospective cohort study	80 children	virus (n = 30) the most common pathogen were rhinovirus, RSV, and adenovirus malaria (n = 27) bacteria (n = 23)	haptoglobin +TNFR-2+ TIMP-1, haptoglobin + IL-10+ TIMP-1, haptoglobin + IL-10, CK-MB

Oved et al, 2015 [41]	prospective cohort study Curiosity	765 children and adults	Training set: viral infections: (n = 167), the most common pathogen were Rhinovirus, Adenovirus, Parainfluenza, Influenza A, and RSV bacterial infections (n = 160) uninfected controls (n = 56) Validation set: viral infections: (n = 167), the most common pathogen were Rhinovirus, Adenovirus, Parainfluenza, Influenza A, and RSV bacterial infections (n = 159) uninfected controls (n = 56)	CRP + TRAIL + IP-10

Eden et al., 2016 [42]	sub-study of prospective cohort study Curiosity	155 children and adults	viral infections: (n = 128) bacterial or mixed infections (n = 27)	CRP + TRAIL + IP-10

Srugo et al., 2017 [43]	prospective double-blind, multicenter study	361 children	unknown (n = 54) viral infections: (n = 239) bacterial infections (n = 68)	CRP + TRAIL + IP-10

van Houten et al., 2017 [44]	prospective, double-blind, international, multicentre study OPPORTUNITY	577 children	unknown (n = 71) viral infections: (n = 435)), the most common pathogen were Rhinovirus and RSV bacterial infections (n = 71)	CRP + TRAIL + IP-10

Ashkenazi-Hoffnung et al., 2018 [45]	sub-study of prospective cohort study Curiosity	314 children and adults	viral infections: (n = 175) bacterial infections (n = 139)	CRP + TRAIL + IP-10

**Table 3:** Studies addressing acute-phase proteins and signaling molecules for the assessment of CAP severity.

Study [Ref.]	Study design	Patients	Mortality (at 28 days follow-up) Prediction rules used for assessing the severity of CAP	Target biomarkers
Park et al., 2012 [49]	prospective observational study	126 adults	survivors (n = 110), non-survivors (n = 16) prediction rules: PSI, CURB65 score, IDSA/ATS guideline PSI class for Survivors: I, II and III (n = 70), IV (n = 28), V (n = 12) PSI class for Non-survivors: I, II and III (n = 1), IV (n = 4), V (n = 11)	PCT, CRP

Kim et al., 2017 [50]	retrospective chart review	125 adults	survivors (n = 112), non-survivors (n = 13) prediction rules: PSI, CURB65 score, IDSA/ATS guideline, APACHE II, SOFA and qSOFA PSI class for Survivors: I, II and III (n = 55), IV or V (n = 57), PSI class for Non-survivors: I, II and III (n = 0), IV or V (n = 13),	PCT, CRP

Kruger et al., 2008 [51]	multicenter prospective observational study CAPNETZ	1671 adults	survivors (n = 1476), non-survivors (n = 70), lost to follow-up (n = 125) CRB-65 score: 0 (n = 557), 1 (n = 608), 2 (n = 275), 3 (n = 58), 4 (n = 10)	PCT, CRP, WBC

Que et al., 2015 [52]	retrospective analysis in small cohort	77 adults	survivors (n = 65), тon-survivors (n = 12) APACHE II, median for survivors: 18, APACHE II, median for non-survivors: 28.5 SAPS II, median for survivors: 42, SAPS II, median for non-survivors: 59.5 SOFA, median for survivors: 8, SOFA, median for non-survivors: 13	PCT, CRP

Haugen et al., 2015 [53]	secondary analysis of data collected in a previously completed randomized double blind, placebo-controlled trial (RCT)	430 children	non-severe CAP (n = 387), severe CAP (n = 43)	27 cytokines: IL-1*β*, IL-1ra, IL-2, IL-4, IL-5, IL-6, IL-7, IL-8/CXCL8, IL-9, IL-10, IL-12 (p70), IL-13, IL-15, IL-17A, bFGF, eotaxin/CCL11, G-CSF, GM-CSF, IFN-*γ*, IP-10/ CXCL10, MCP-1/CCL2, MIP-1*α*/CCL3, MIP-1*β*/CCL4, RANTES/CCL5, TNF-*α*, PDGF-BB and VEGF

Wang et al., 2013 [54]	prospective observational study	121 adults	CAP patients (n = 61), healthy controls (n = 60) prediction rules: PSI, CURB65 score, APACHE II	CRP, WBC, YKL-40

Spoorenberg et al., 2018 [55]	secondary analysis of data collected in a previously completed randomized controlled trial (RCT)	311 adults	survivors (n =272) non-survivors (n = 19), healthy controls (n = 20) PSI class: I - III (n = 152), IV - V (n = 139)	SP-D, YKL-40, CCL18, CA 15-3, IL-6, CRP

Wang et al., 2018 [57]	prospective observational study	102 adults	mild CAP (n = 36), severe CAP (n = 35), healthy controls (n = 31) prediction rule: CURB-65 score	IRF5, IFN-a, IL-6, IL-10, IP10, TNF-a mRNA levels of IRF5, IL-6, IL-10, IP10, TNF-a, and IFN-a in peripheral blood and bronchoalveolar lavage fluid

Abbreviations:

CURB65 score (confusion, uremia, respiratory rate, blood pressure, age 65 years),

PSI - the pneumonia severity index,

IDSA/ATS guidelines - the Infectious Disease Society of America (IDSA) and the American Thoracic Society (ATS) issued guidelines,

SAPS II - the Simplified Acute Physiology Score II,

APACHE II - the Acute Physiology and Chronic Health Evaluation II score,

SOFA - the Sepsis-related Organ Failure Assessment score,

qSOFA - quick SOFA.

**Table 4:** Candidate biomarkers associated with CAP severity.

Study [Ref.]	Study design	Patients	Mortality (at 28 – 30 days follow-up) Prediction rules used for assessing the severity of CAP	Target proteins
Christ-Crain et al, 2006 [59]	prospective observational study	302 adults	survivors (n = 264), non-survivors (n = 38) PSI class: I, II and III (n = 120), IV (n = 130), V (n = 52)	mid regional proADM, CRP, PCT, WBC

Kruger et al., 2010 [60]	multicenter prospective observational study CAPNETZ	728 adults	survivors (n =691), non-survivors (n =37): within 28 days (n = 18), and within 180 days (n = 19) CRB-65 score: 0 (n = 306), 1 (n = 317), 2 (n = 90), 3 (n = 15), 4 (n = 0)	mid regional proADM, mid regional proANP, copeptin, CT-ET-1, PCT, CRP

Espana et al., 2015 [61]	prospective observational study	491 adults	survivors (n = 456), non-survivors (n = 35) PSI I-III (n = 325), CURB-65 score 0-1 (n = 284), SCAP score 0-1 (n = 353)	proADM, PCT, CRP

Gutbier et al., 2018 [62]	multicenter prospective observational study CAPNETZ prospective observational ancillary study of CAPNETZ and PROGRESS	148, and 395 adults (independent cohorts)	cohort 1 (CAPNETZ): survivors (n = 74), non-survivors (n = 74); CRB-65 score: 0 (n = 8), 1 (n = 58), 2 (n = 66), 3 (n = 16), 4 (n = 0) cohort 2 (PROGRESS): survivors (n = 376), non-survivors (n = 19); CRB-65 score: 0 (n = 130), 1 (n = 124), 2 (n = 90), 3 (n = 39), 4 (n = 11), 5 (n = 1)	Ang-1, Ang-2

Dai et al., 2015 [63]	retrospective analysis in small cohort	52 children	not reported	LPS

Huang et al., 2014 [65]	proteomic study to identify and validate markers	390 children (identification) 293 children (validation)	cohort 1 (identification): non-severe CAP (n = 96), severe CAP (n = 76), very severe CAP (n = 32), healthy controls (n = 186). cohort 2 with respiratory distress (validation): pneumonia (n = 238), malaria (n = 41), pneumonia and malaria (n = 14)	NGAL/Lpc-2, haptoglobin CRP, vWF

Bacci et al., 2015 [66]	prospective observational study in small cohort	27 adults	survivors within 7 days (n =19), non-survivors within 7 days (n = 7): survivors within 28 days (n = 16), non-survivors within 28 days (n = 10) prediction rules CURB-65 score and CRB score: cohort data not reported	IL-1, IL-6, TNF-a, CRP, homocystein

Kim et al., 2016 [67]	prospective observational study	362 adults	survivors (n = 324), non-survivors (n = 38) PSI class: I, II and III (n = 186), IV (n = 128), V (n = 48)	NGAL/Lpc-2, PCT

Nikaido 2015 [69]	prospective observational study in small cohort	41 adults	A-DROP score: mild pneumonia A-DROP 0 – 1 (n = 17), severe pneumonia A-DROP 2–5 (n = 13), healthy volunteers (n = 11)	Syndecan-4 (SYN4)

Esposito et al, 2016 [70]	prospective observational study	110 children	severe CAP (n = 84)	NGAL/Lpc-2, SYN4, CRP

Abbreviations:

SCAP - severe community-acquired pneumonia score,

CURB65 score (Confusion, Uremia, Respiratory rate, Blood pressure, age 65 years),

A-DROP scoring system (Age, Dehydration, Respiratory rate, Orientation, blood Pressure).
